# Dark septate endophytes promote the growth of *Cynodon dactylon* under drought stress and enhance its potential for use in the ecological restoration of slopes

**DOI:** 10.3389/fpls.2025.1537256

**Published:** 2025-10-21

**Authors:** Haoji Jia, Qiming Geng, Mingyi Li, Ran Wang, Fuhao Wang, Yuxin Deng, Wennian Xu, Daxiang Liu

**Affiliations:** ^1^ Key Laboratory of Geological Hazards on Three Gorges Reservoir Area (China Three Gorges University), Ministry of Education, Yichang, China; ^2^ Hubei Provincial Engineering Research Center of Slope Habitat Construction Technique Using Cement-based Material (China Three Gorges University), Yichang, China; ^3^ College of Biological & Pharmaceutical Sciences, China Three Gorges University, Yichang, China

**Keywords:** drought, vegetation concrete, dark septate endophytes, *Cynodon dactylon*, osmoregulation substance, antioxidant enzyme activities

## Abstract

**Introduction:**

The objective of this study was to investigate whether dark septate endophytes (DSEs) can increase plant drought tolerance in the context of vegetation concrete, which is a complex environment.

**Methods:**

This study employed a controlled simulation experiment to investigate the influence of inoculation with diverse DSEs, namely, *Paraphoma chrysanthemicola* (PC), *Alternaria alternata* (AA), and *Cladosporium cladosporioides* (CC), on the growth, photosynthetic characteristics, osmoregulatory substance content, and antioxidant enzyme activities of *Cynodon dactylon* in vegetation concrete subjected to drought stress.

**Results:**

These findings demonstrated that DSEs were capable of effectively mitigating the adverse impacts of drought on plant growth. Under moderate drought (MD 55%±5% of the maximum moisture capacity in the field), DSEs increased the dry weight (DB), net photosynthetic rate (Pn), soluble sugar (SS) and peroxidase (POD) of *C. dactylon* by up to 14.21%, 32.63%, 40.73% and 31.43%, respectively, and reduced the malondialdehyde (MDA) content by 8.02-13.77%. Furthermore, under severe drought (SD, 35%±5% of the maximum moisture capacity in the field), DSE inoculation enhanced the photosynthetic capacity of *C. dactylon*, stimulated the accumulation of osmoregulatory compounds such as proline (Pro) and soluble protein (SP), and mitigated the water loss associated with drought.

**Conclusion:**

The results demonstrate that DSE inoculation enhances the drought resistance of plants used in vegetation concrete by increasing the photosynthetic rate, and contents of antioxidant enzymes and osmoregulatory substances. This study provides reference for the use of DSEs in ecological restoration with vegetation concrete.

## 1 Introduction

Drought represents one of the most significant and pervasive natural disasters worldwide. Due to global warming, the frequency, intensity and spatial distribution of droughts have increased markedly, posing a substantial threat to food and ecological security and becoming a primary factor limiting the sustainable development of social and ecological systems (Cheng et al., 2024; Feng et al., 2024). The role of vegetation in mitigating climate change is significant, because of its capacity for carbon sequestration (He T. et al., 2023). Nevertheless, the accelerated pace of large-scale construction projects inevitably exerts considerable influence on the surrounding ecological environment (Gan et al., 2018). In particular, the damage to slopes created by earthmoving excavation has resulted in the destruction of original topography and vegetation. This results in a significant loss of habitat conditions, which in turn leads to a reduction in biodiversity and an increase in soil erosion and other problems. It is challenging to restore the original appearance by relying only on natural restoration techniques. Traditional slope protection methods tend to prioritize mechanical stability, but they lack the ecological functions of landscape and self-repair. This makes it difficult to achieve the restoration or reconstruction of the vegetation cover (Chen et al., 2022; Fang et al., 2022; Ganapathy et al., 2024; Tudorica and Bob, 2024). Consequently, there has been a notable increase in the utilization of ecological slope protection methods, including the vegetation concrete technique (Shen et al., 2023). This involves the mixing of soil, cement, organism of habitat material, amendment of habitat material and vegetative seeds in specific proportions that are determined, by the characteristics of the slopes in the restoration area and the landscape needs. The resulting mixture is then laid on the slopes using barbed wire and fixed anchors and sprayed onto the slopes using sprayers. This process facilitates regreening and slope protection (Yang et al., 2019). This approach has facilitated the large-scale application of vegetation concrete, which provides engineering protection and supports ecological restoration, and has the potential to be sustainable and to sequester carbon (Cheng et al., 2020; Zhao et al., 2022; Xu et al., 2024). However, at present, slope reinforcement and vegetation restoration cannot be conducted concurrently in dry areas.

In recent years, the majority of research conducted on vegetation concrete has focused on enhancing its physico-mechanical strength through the incorporation of external admixtures and improving its fertility characteristics (Huang et al., 2021; Liu D. et al., 2023). This research has predominantly been concentrated in alpine areas (Yang Y. et al., 2023) and regions that experience heavy rainfall (Li M.Y. et al., 2022), with comparatively less research in arid zones. In the initial phase of vegetation concrete restoration of steep slopes, the elevated and precipitous topography gives rise to the formation of surface runoff on the slope surface due to insufficient infiltration of water. Furthermore, plants are susceptible to drought stress due to the evapotranspiration effect of the substrate (He J. et al., 2023). Drought impedes plant root growth and development. In response to drought stress, plants reduce their root and stem lengths and decrease their biomass (Bhandari et al., 2023). The persistence of drought stress has the capacity to alter the morphological structure of plants, as well as the structure of plant chloroplasts. This results in a reduction in chlorophyll content and the closure of plant stomata, in addition to a decline in the net photosynthetic rate, transpiration rate and stomatal conductance of plant leaves. This, in turn, has a direct impact on photosynthetic efficiency (Luan et al., 2023; Zhang et al., 2023; Wang et al., 2024). Concurrently, drought exerts a detrimental impact on soil structure, reducing soil productivity and restricting plant water and nutrient uptake. Additionally, it impairs reactive oxygen species metabolism and membrane lipid fluidity in plants, which in turn precipitates peroxidation of plant cell membrane lipids, osmosis, and redox imbalance, ultimately leading to plant death (Wang et al., 2022; Lei et al., 2023; Yang W. et al., 2023). These issues are particularly prevalent in the ecological restoration of slopes. The implementation of input control methods, such as the establishment of irrigation systems and replanting measures, can temporarily alleviate the aforementioned stresses (Tamura et al., 2017; Strack and Stoll, 2022). However, these measures lack long-term efficacy in improving and restoring plant nutrient utilization and ecosystem self-sustainability. Consequently, the deterioration of ecosystems persists, often accompanied by high maintenance costs and significant resource inputs (Guo et al., 2024). Recently, plant–microbe symbiosis has received widespread attention and compared with traditional ecological restoration techniques, microorganisms have the potential to establish beneficial symbiotic relationships with plants, thereby mitigating the adverse effects of environmental stressors on plant health (Gehring et al., 2020; Abideen et al., 2022; Chieb and Gachomo, 2023). Thus, this approach provides a promising new strategy for improving the drought tolerance of plants used in the ecological restoration of steep slopes and for improving the efficiency and sustainability of ecological restoration projects on steep slopes.

Symbiotic fungi play a significant role in ecological processes (He et al., 2024), as evidenced by the effectiveness of root-associated endophytic fungi in enhancing plant performance and drought resistance (Lu et al., 2021; He et al., 2022a). The term “dark septate endophytes” (DSE) is used to describe a group of small soil fungi that colonize plant roots. These fungi have dark-colored mycelia and distinctive septa, which widely colonize plant root cells and are capable of forming “microsclerotia” structures (Liu and Wei, 2019). The extended mycelia of DSEs are capable of modifying the morphology and structure of plant roots, expanding the root network and enhancing the water uptake of host plants under drought stress (Li et al., 2024). Liu N. et al. (2022) observed that the incorporation of a DSE inoculant led to a notable increase in seedling biomass and the activities of drought-resistant enzymes. Under drought stress, DSE inoculation has been demonstrated to reinforce the resilience of plant root cells; safeguard the structural integrity of cell membranes, nuclei, mitochondria, and other organelles; and mitigate the adverse effects of drought stress on these organelles (Liu and Wei, 2019). Furthermore, the colonization of plants by DSEs has been show to stabilize photosynthetic parameters, reduce stomatal conductance and decrease overall water loss (Li M. et al., 2022). Numerous studies have demonstrated that DSEs play a role in regulating the endogenous hormone content of host plants, as well as increasing the secretion of proline, superoxide dismutase and catalase. Furthermore, it has been observed that DSEs support the synthesis of melanin under stressful conditions by binding free oxygen, thereby protecting cell membrane stability and enhancing plant resistance to environmental stresses, including drought (He et al., 2022b; Bi and Xue, 2023; Li W. et al., 2023).

The impact of drought on the ecological restoration of slopes is significant, resulting in adverse effects on the normal growth and development of plants used for slope protection. DSEs have been demonstrated to increase drought resistance in host plants, thereby conferring resilience to plants growing in areas experiencing desertification and drought (Li X. et al., 2022) and increasing the productivity of medicinal and agricultural crops (Han et al., 2021; Qu et al., 2022). Nevertheless, DSEs are less frequently employed for slope ecological protection; thus, this study proposes the introduction of DSEs into the domain of vegetation concrete. By simulating drought stress, this study investigated the effects of DSEs on the physiological and ecological characteristics of Cynodon dactylon in vegetation concrete under drought conditions. The aim was to clarify the mechanisms through which DSEs increase the drought tolerance of slope-stabilizing plants to provide new solutions and theoretical foundations for improving the drought tolerance of these plants and advancing the ecological restoration process of vegetation concrete.

## 2 Materials and methods

### 2.1 Experimental design

The DSE strain *Paraphoma chrysanthemicola* (PC) was obtained from the China General Microbiological Culture Collection Center (CGMCC), while the *Alternaria alternata* (AA) and *Cladosporium cladosporioides* (CC) strains were sourced from the China Center of Industrial Culture Collection (CICC). The seeds of the test plant species, namely *C. dactylon*, were provided by the Vegetation Concrete Slope Protection Project.

In this study, a two-factor test was employed, with factor (1) representing different soil water treatments: well-watered (WW 75%±5% of the maximum moisture capacity in the field), moderate drought (MD 55%±5% of the maximum moisture capacity in the field) and severe drought (SD 35%±5% of the maximum moisture capacity in the field) (Sheng et al., 2023). Factor (2) represented the different DSE inoculation treatments, namely *Paraphoma chrysanthemicola* (PC), *Alternaria alternata* (AA), *Cladosporium cladosporioides* (CC) and a control without inoculation (CK). A total of 12 treatments were used in the experiment, with six replicates per treatment, resulting in a total of 72 pots.

### 2.2 Test material handling and test conditions

The test site was located at the Hubei Research Centre for Cement-based Ecological Rehabilitation Technology, which is located at China Three Gorges University in Yichang city, Hubei Province, China. The potting test was conducted in accordance with the current “Technical Code for Eco-restoration of Vegetation Concrete on Steep Slope of Hydropower Projects” (National Energy Administration of the People’s Republic of China, 2016) with the objective of formulating the vegetation concrete. The prepared vegetation concrete was then placed in pots with a top diameter of 20 cm, a base diameter of 15 cm, and a height of 14 cm. The base layer (devoid of seeds) was paved first, followed by the addition of the surface layer (containing seeds). Each pot was filled with 3 kg of vegetation concrete.

**Figure 1:**
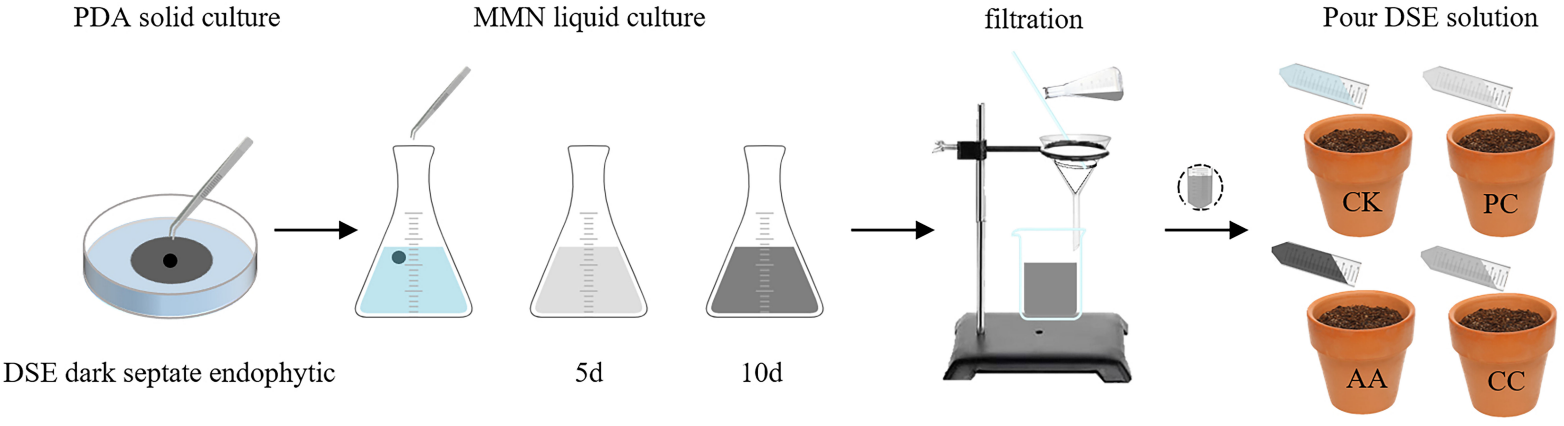
Illustration of the process followed to obtain fungal extract.

The fungal mixture was prepared by placing the cultivated DSE fungal mixture in Modified Melin Norkrans (MMN) medium and incubating it in a constant temperature shaker. The fungal mixture was prepared at 27 °C and 120 r/min for 10 days, after which the mycelium was filtered, and the solution was removed. Each pot was filled with 40 mL of solution, and a control was inoculated with 40 mL of sterilized MMN medium culture solution (Xie et al., 2021) as shown in **Figure 1**. Following a 30-day period of vegetation concrete maintenance and normal plant growth, drought stress treatment was initiated using the weighing method (Qi et al., 2023). Measurements were taken daily at 6 p.m., and the soil moisture content was regulated through artificial irrigation, ensuring that the relative soil moisture content remained within the parameters defined by the experimental design. After 30 days of drought treatment, the factors were tested.

### 2.3 Determination of the growth pattern

Following a 30-day period of drought stress, plant height was measured directly using the straightedge method. Nine replicates were selected for each treatment, comprising three representative and comparable plants from the selected pots. For biomass determination, three pots were selected for each treatment, and all the plants in the pots were removed intact and placed in sample bags. The sample bags were placed in an oven at 105 °C for 30 minutes and then dried at 75 °C to a constant weight, and the resulting weight was taken as the dry weight.

### 2.4 Measurements of photosynthetic parameters

The net photosynthetic rate (Pn), stomatal conductance (Gs), intercellular CO_2_ concentration (Ci) and transpiration rate (Tr) of the functional leaves were determined using the LI-6400 portable photosynthesis system. A fixed light intensity of 1000 μmol m^−2^ s^−1^ was employed, and three replicate sample of each treatment were collected on sunny mornings, i.e., from 9:00 am to 11:00 am after 30 days of stress.

### 2.5 Determination of antioxidant enzyme activities

The activities of the antioxidant enzymes superoxide dismutase (SOD), peroxidase (POD) and catalase (CAT) were determined in accordance with the methodology described by Su et al. (2012). Fresh plant leaves weighing 0.5 g were pulverized in a solution of 50 mmol/L phosphate buffer (pH 7.8), containing 0.2 mmol/L EDTA, 1% PVP and 5 mmol/L MgCl_2_ that had been cooled in a container of ice. The homogenate was subjected to centrifugation at 12,000 × g for 20 minutes at 4 °C. The resulting supernatant was subsequently analysed to determine the activity of the antioxidant enzymes. SOD activity was determined using the nitro-blue tetrazolium (NBT) photoreduction method, POD activity was measured using the oxidation of guaiaco method, and CAT activity was measured by ultraviolet absorption, with three replicates conducted for each treatment.

### 2.6 Determinations of the content of osmoregulatory substances

The MDA content was determined by the thiobarbituric acid method (Rady et al., 2020). The soluble protein (SP) content was determined via the Kh Khao Maas Brilliant Blue G-250 colourimetric method (Ahmed et al., 2020). The soluble sugar (SS) content was determined by the anthrone method (Li et al., 2024), and the proline (Pro) content was determined by the ninhydrin colourimetric method (Bates et al., 1973). Three replicates were performed for each treatment.

### 2.7 Statistical analyses

This study included nine replications for each group of treatments with respect to plant height, with three replications carried out for the remaining parameters. The resulting data are expressed as the means ± standard errors. Data were organized using Microsoft Excel 2019, while analysis of variance (ANOVA) was performed using SPSS Statistics 26. The plotting and Person correlation, as well as the PCA, were carried out using Origin 2024.

## 3 Results

### 3.1 Plant height and dry weight

**Figure 2:**
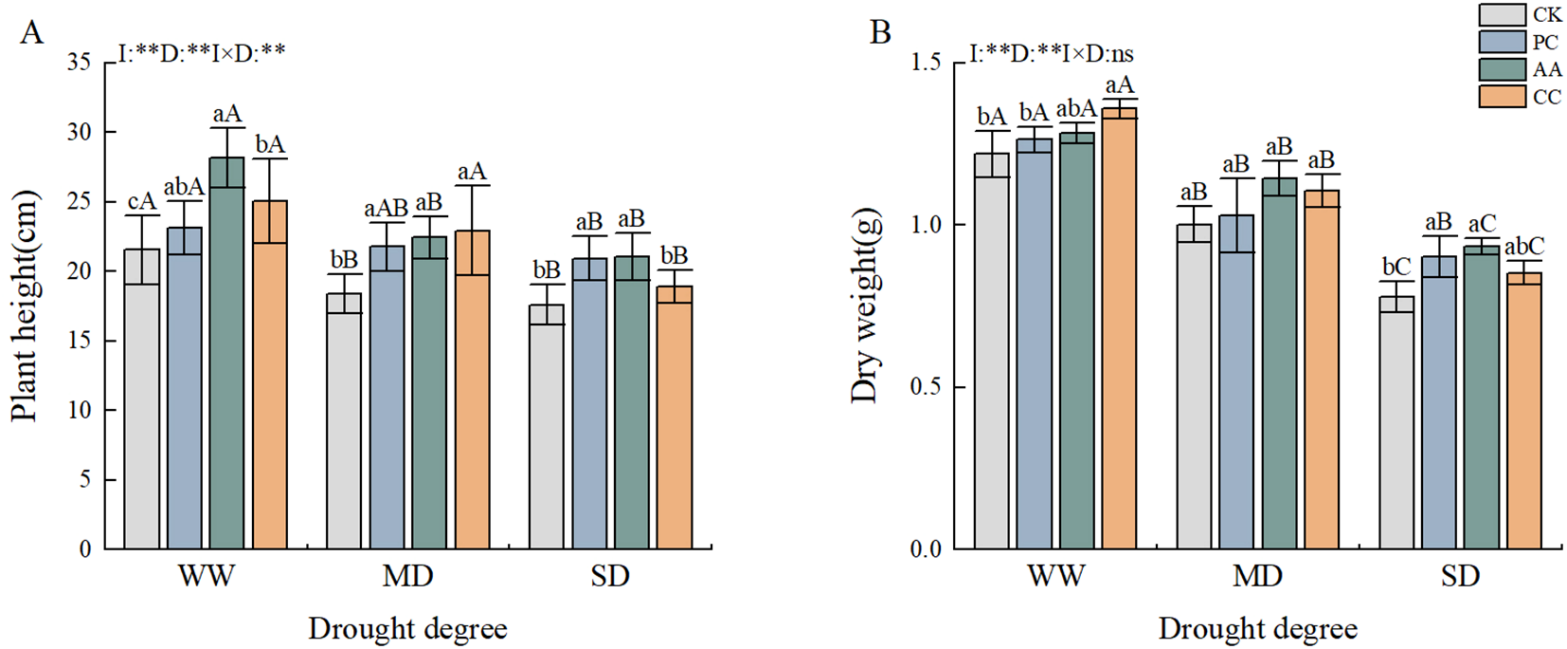
Effects of DSEs on the height and dry weight of *Cynodon dactylon* under drought conditions. **(A)** Plant height, **(B)** Dry weight (DW). Different lowercase letters indicate significant differences between different DSE inoculations under the same drought conditions (p<0.05). Different uppercase letters indicate significant differences between different drought conditions under the same inoculation with DSE (p<0.05). WW, well-watered; MD, moderate drought; SD, severe drought. I, inoculated with DSEs; D, degree of drought; I×D, interaction between inoculation with DSEs and drought stress. **p<0.01; ns, not significant. CK, control without inoculation; PC, treatment with inoculation of Paraphoma chrysanthemicola; AA, treatment with inoculation of Alternaria alternata; CC, treatment with inoculation of Cladosporium cladosporioides.

The drought and DSE treatments had highly significant effects on the plant height and dry weight of *C. dactylon* (**Figure 2**). The height and dry weight of *C. dactylon* tended to decrease with increasing drought stress. Compared with those in the CK treatment, the plant height and dry weight of *C. dactylon* in the CC treatment notably increased by 16.24% and 11.51%, respectively. Compared with those under the CK treatment, the plant height and dry weight under the AA treatment increased by 19.56–30.67% and 5.34–20.00%, respectively. Moreover, a significant difference was observed under severe drought conditions.

### 3.2 Photosynthetic attributes

**Figure 3:**
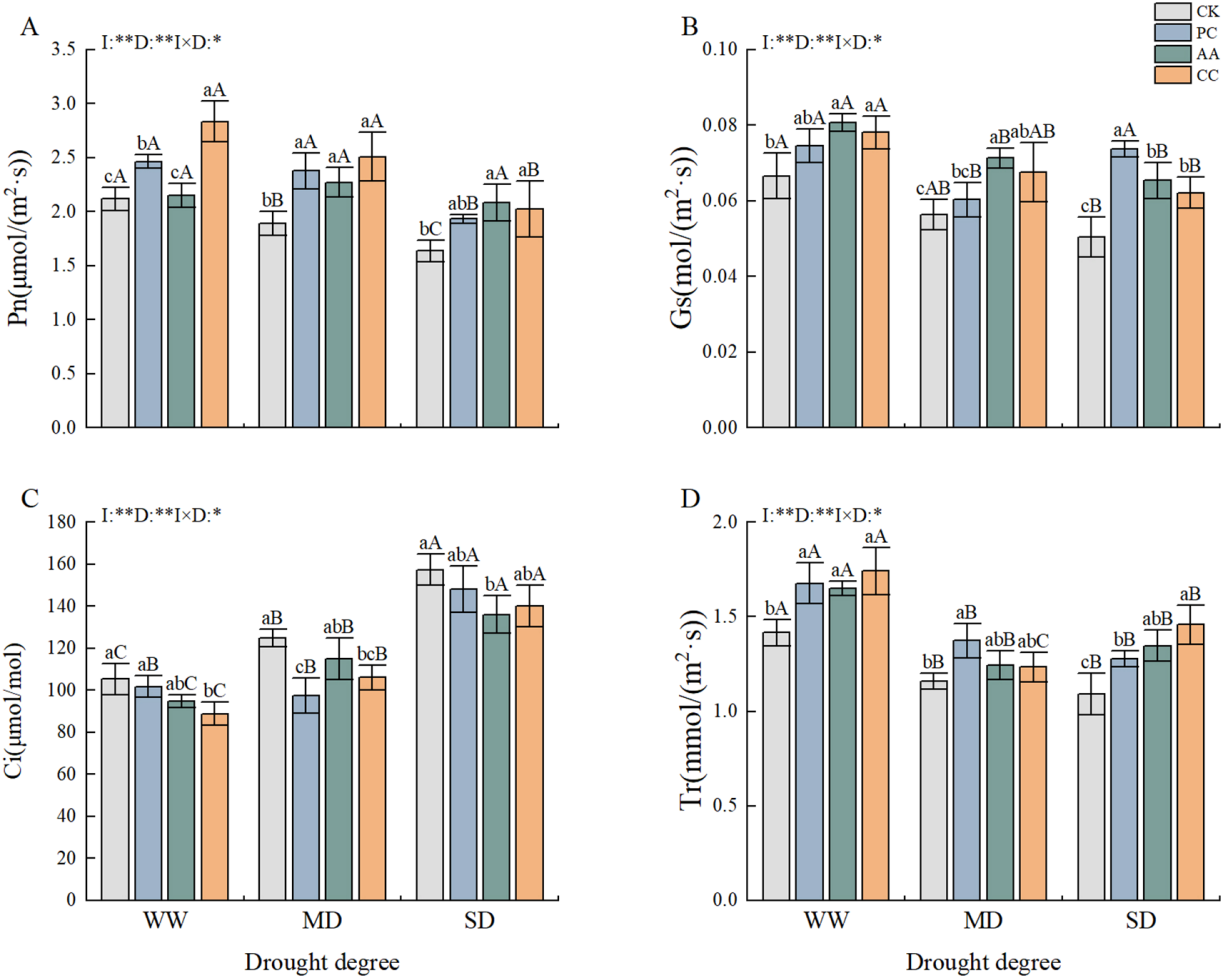
Effects of DSEs on the photosynthetic characteristics of *Cynodon dactylon* under drought conditions. **(A)** Net photosynthetic rate (Pn), **(B)** Stomatal conductance (Gs), **(C)** Intercellular CO_2_ concentration (Ci), **(D)** Transpiration rate (Tr). Different lowercase letters indicate significant differences between different DSE inoculations under the same drought conditions(p<0.05). Different uppercase letters indicate significant differences between different drought conditions under the same inoculation with DSE (p<0.05). WW, well-watered; MD, moderate drought; SD, severe drought. I, inoculated with DSEs; D, degree of drought; I×D, interaction between inoculation with DSEs and drought stress. *p<0.01; **p<0.01. CK, control without inoculation; PC, treatment with inoculation of Paraphoma chrysanthemicola; AA, treatment with inoculation of Alternaria alternata; CC, treatment with inoculation of Cladosporium cladosporioides.

The interaction between drought and DSEs had a marked effect on the photosynthetic attributes of *C. dactylon* (**Figure 3**). Overall, the Pn of *C. dactylon* tended to decrease as the degree of drought stress increased. The maximum level of plant Pn was reached under the well-watered and moderate drought CC treatments, which resulted in significant increases of 33.85% and 32.63%, respectively, in comparison with that of the CK. Conversely, the AA treatment under severe drought demonstrated even greater efficacy, with an increase in plant Pn of 27.49% in comparison with that in the CK (**Figure 3A**). As drought stress intensified, the changes in Gs and Pn in *C. dactylon* remained consistent. In the well-watered and moderate drought treatments, the plant Gs was greater in the PC treatment group than in the CK group, yet the difference was not significant. However, under severe drought, the PC, AA, and CC treatments presented significantly greater values than did the CK treatment, with values of 46.17%, 29.52%, and 23.18%, respectively (**Figure 3B**).

Unlike the Pn and Gs, the Ci of *C. dactylon* tended to increase with the degree of drought stress. However, this effect was mitigated by different DSE treatments. Compared with that under the CK treatment, the Ci under both the normal moisture and moderate drought CC treatments were reduced in all the groups. The greatest reduction, amounting to 15.61%, was observed in the normal moisture group. In contrast, the results of the AA treatment under severe drought conditions were notably different from those of the CK treatment, with a 13.56% decrease (**Figure 3C**). Compared with the CK treatment, the application of treatments with different strains resulted in an increase in the Tr of *C. dactylon*. The maximum Tr levels observed under the well-watered and severe drought conditions were recorded in the CC treatment, with 23.03% and 33.64% increases, respectively, compared with those in the CK. Conversely, under moderate drought conditions, the highest plant Tr was observed in the PC treatment group, with an 18.49% increase compared with that in the CK group (**Figure 3D**).

### 3.3 Osmoregulation

**Figure 4:**
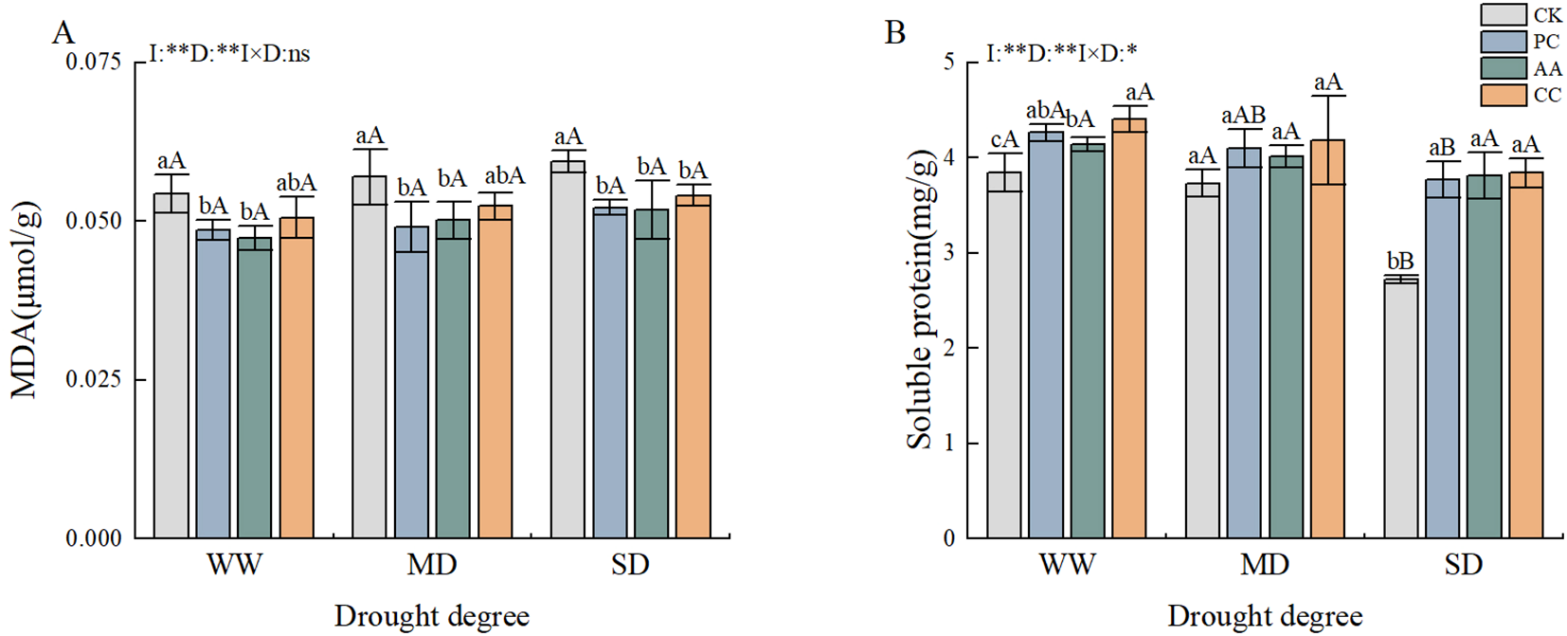
Effects of DSEs on the MDA and SP contents of *Cynodon dactylon* under drought conditions. **(A)** Malondialdehyde (MDA), **(B)** Soluble protein (SP). Different lowercase letters indicate significant differences between different DSE inoculations under the same drought conditions(p<0.05). Different uppercase letters indicate significant differences between different drought conditions under the same inoculation with DSE (p<0.05). WW, well-watered; MD, moderate drought; SD, severe drought. I, inoculated with DSEs; D, degree of drought; I×D, interaction between inoculation with DSEs and drought stress. *p<0.05; **p<0.01; ns, not significant. CK, control without inoculation; PC, treatment with inoculation of Paraphoma chrysanthemicola; AA, treatment with inoculation of Alternaria alternata; CC, treatment with inoculation of Cladosporium cladosporioides.

The effects of the drought and DSE treatments on the malondialdehyde (MDA) and SP contents of *C. dactylon* were highly significant, with the interaction between the two treatments also having significant effects on the SP content (**Figure 4**). In accordance with the prevailing drought conditions, the MDA content of *C. dactylon* markedly decreased in comparison with that in the CK when the plants were subjected to the AA and CC treatments. The MDA content was significantly lower under the severe drought conditions in the PC, AA, and CC treatment groups than in the CK, with reductions of 12.13%, 12.75%, and 8.89%, respectively (**Figure 4A**). These findings suggest that the cell membrane lipid peroxidation caused by drought was somewhat alleviated by DSE inoculation. Unlike the MDA content, the SP content of *C. dactylon* gradually decreased in response to increasing drought stress. However, the SP content of *C. dactylon* was greater in the DSE treatment than in the CK treatment. Under both the well-watered and severe drought conditions, the SP content associated with each DSE strain was significantly greater than that under the CK treatment. However, no significant differences were evident among the moderate drought treatments (**Figure 4B**).

**Figure 5:**
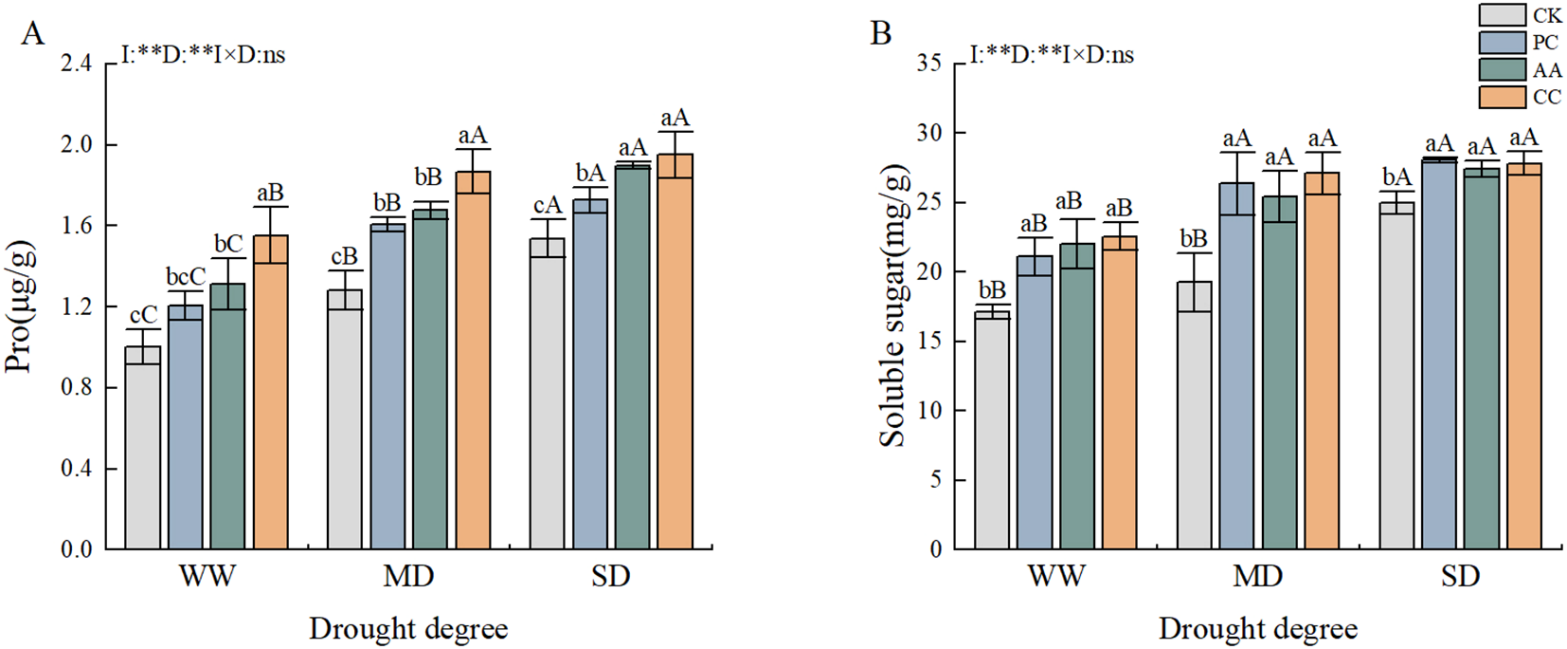
Effects of DSEs on the Pro and SS content of *Cynodon dactylon* under drought conditions. **(A)** Proline (Pro), **(B)** Soluble sugar (SS). Different lowercase letters indicate significant differences between different DSE inoculations under the same drought conditions (p<0.05). Different uppercase letters indicate significant differences between different drought conditions under the same inoculation with DSE (p<0.05). WW, well-watered; MD, moderate drought; SD, severe drought. I, inoculated with DSEs; D, degree of drought; I×D, interaction between inoculation with DSEs and drought stress. **p<0.01; ns, not significant. CK, control without inoculation; PC, treatment with inoculation of Paraphoma chrysanthemicola; AA, treatment with inoculation of Alternaria alternata; CC, treatment with inoculation of Cladosporium cladosporioides.

The drought and DSE treatments had highly significant effects on the Pro and SS contents of *C. dactylon*. The Pro and SS contents under each DSE treatment were proportional to the intensity of drought stress (**Figure 5**). The Pro content remained high under the DSE treatment. The Pro content of *C. dactylon* increased significantly under severe drought conditions, and all the DSE treatments resulted in an increase in the Pro content compared with that in the CK, with the greatest increase in the CC treatment, in which the content was 27.02% greater than that in the CK treatment (**Figure 5A**). The application of DSE resulted in a notable increase in the SS content within *C. dactylon*. The most pronounced increase was observed in the CC treatment under moderate drought, which resulted in a significant 40.73% increase in the SS content relative to that in the CK. In contrast, the AA treatment under severe drought conditions resulted in the least pronounced increase in the SS content compared to that in the CK (9.83%) (**Figure 5B**).

### 3.4 Antioxidant enzymes

**Figure 6:**
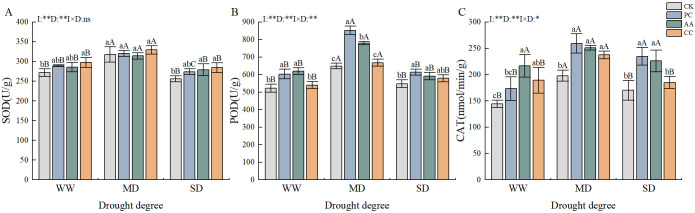
Effect of DSEs on antioxidant enzyme activities of *Cynodon dactylon* under drought conditions. **(A)** Superoxide dismutase (SOD), **(B)** Peroxidase (POD), **(C)** Catalase (CAT). Different lowercase letters indicate significant differences between different DSE inoculations under the same drought conditions (p<0.05). Different uppercase letters indicate significant differences between different drought conditions under the same inoculation with DSE (p<0.05). WW, well-watered; MD, moderate drought; SD, severe drought. I, inoculated with DSEs; D, degree of drought; I×D, interaction between inoculation with DSEs and drought stress. *p<0.05; **p<0.01; ns, not significant. CK, control without inoculation; PC, treatment with inoculation of Paraphoma chrysanthemicola; AA, treatment with inoculation of Alternaria alternata; CC, treatment with inoculation of Cladosporium cladosporioides.

The antioxidant enzyme activities of *C. dactylon* initially increased but then decreased in response to increasing drought stress. The interaction effect between drought and DSEs had a highly significant effect on the POD activity of *C. dactylon* (**Figure 6**). The SOD content of *C. dactylon* significantly increased by 9.54% and 11.14% under the well-watered and severe drought CC treatments, respectively; however, no significant differences were detected among the treatments under moderate drought (**Figure 6A**). The POD activity of *C. dactylon* under moderate drought was significantly greater than that under the well-watered and severe drought treatments. The highest POD activity was recorded in the PC treatment under moderate drought conditions, which presented a significant increase of 31.43% compared with that in the CK treatment (**Figure 6B**). The CAT activity of *C. dactylon* in the DSE treatment under moderate drought conditions was significantly increased by 30.97%, 26.79% and 20.00% in the PC, AA and CC treatments, respectively, compared with that in the CK treatment. Furthermore, the AA treatment resulted in significantly elevated CAT activity compared with that in the CK treatment under the watered and severe drought conditions (**Figure 6C**). The results demonstrated that the DSE-inoculated *C. dactylon* presented elevated antioxidant enzyme activities.

### 3.5 Principal component analysis and correlation analysis

**Figure 7:**
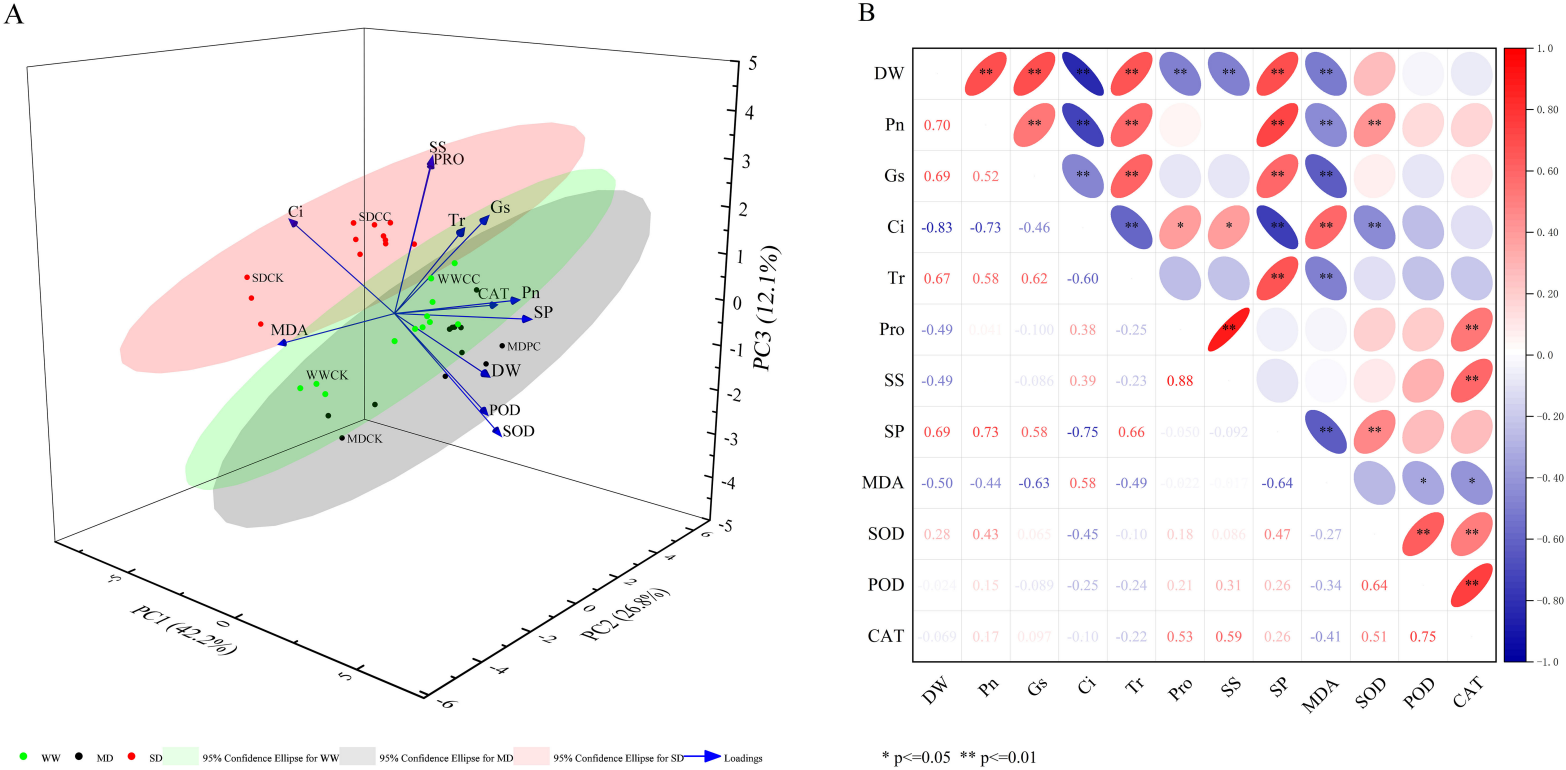
Principal component analysis (PCA) and correlation analysis of Cynodon dactylon following inoculation with DSEs under different drought conditions. **(A)** Principal component analysis (PCA), **(B)** Correlation analysis. *p<=0.05; **p<=0.01.

A principal component analysis of 12 physiological indicators, including DW, CAT, and Pro in *C. dactylon* under various treatments, revealed three significant principal components (PCs) with eigenvalues greater than 1, collectively accounting for 81.1% of the total variance. The first principal component (PC1) was influenced primarily by DW, SP, Pn, Ci, and MDA, indicating that photosynthesis and the level of cellular membrane lipid peroxidation in *C. dactylon* under drought stress may serve as key indicators of the severity of plant damage due to drought. The second principal component (PC2) was influenced by CAT, POD, SS, and Tr, suggesting that antioxidant enzymes in *C. dactylon* may play a significant role in the ability of plants to tolerate drought stress. The third principal component (PC3) was determined mainly by Pro, Gs, and SOD (**Figure 7A**).

A correlation was established between twelve physiological indices of treated *C. dactylon*. The results demonstrated that the DW of *C. dactylon* was highly significantly positively correlated with Pn, Gs, Tr and SP (p < 0.01) and highly significantly negatively correlated with Ci, Pro, MDA and SS (p < 0.01). These findings corroborate the results of previous analyses,, further substantiating that the elevated MDA content resulting from drought stress intensified membrane lipid peroxidation in *C. dactylon* cells, thereby impeding plant growth. In response, *C. dactylon* increased the contents of Pro and SS through its own metabolism to sustain normal growth and development. Furthermore, the increase in photosynthesis positively contributed to the accumulation of *C. dactylon* biomass (**Figure 7B**).

## 4 Discussion

Drought is one of the most significant environmental factors limiting the growth and development of plants. The development of a community of endophytic fungi and host plants represents a significant strategy for enhancing the resilience of host plants (Pang et al., 2020; Lu et al., 2023; Gu et al., 2024). The present study revealed a significant reduction in both the height and biomass of *C. dactylon* under drought conditions. However, compared to the CK, inoculation with DSEs, promoted the plant height and biomass of *C. dactylon*. This may be attributed to the inhibition of plant cell division under drought, which ultimately results in the retardation of plant growth and development (Ganie and Ahammed, 2021). The mycorrhizal symbionts formed by inoculation with DSEs function as bridges between the host plant and the soil, increasing the contact area between the plant root system and the soil and facilitating water uptake by the plant (Li M. et al., 2022). Furthermore, DSEs facilitate the mineralization of organic matter and the conversion of insoluble phosphates in the soil through the secretion of particular enzyme metabolites, including proteases and xylanases (Luo et al., 2023). The mycelium of DSEs around the root system of a plant assists in the retention of water and the transportation of nutrients to the interior of the plant, promote the uptake of nutrients by the plant (Ruotsalainen et al., 2022). They assist host plants by increasing their capacity to endure drought stress and further promote plant growth.

The process of photosynthesis serves as the foundation for plant growth and development, as well as the accumulation of materials (Wang et al., 2022). Photosynthesis is a fundamental process that directly affects plant growth, with water being one of its primary influencing factors. The present study demonstrated that the Pn of *C. dactylon* significantly decreased under drought conditions, with the magnitude of this decrease increasing in conjunction with the intensification of drought stress. Concurrently, the Ci of the plant leaves increased as the Pn and Tr decreased. Consequently, this study concluded that non-stomatal factors played a pivotal role in limiting photosynthesis in response to drought These findings are consistent with those reported by Liu et al. (2024). At this juncture, the chloroplast structure was irreversibly compromised, which directly resulted in the weakening of the light reaction process and a decrease in the photosynthetic rate (Li Y. et al., 2023). Compared with the Pn values of plants that had not undergone DSE inoculation, the Pn of the treated *C. dactylon* plants was sustained at a high level following inoculation with DSE, and the Gs and Tr also increased, which indicated that inoculation with DSE increased the photosynthetic capacities of the plants. The increase in leaf Gs and Tr under DSE inoculation facilitated the transport and conduction of plant nutrients, thereby enabling the plant to obtain a greater quantity of nutrients (Miranda et al., 2023). Furthermore, the increase in Tr regulated the surface temperature of the plant leaves, preventing overheating and reducing the damage caused by drought stress. These findings indicate that inoculation with DSEs may regulate plant water use efficiency and increase resistance to drought. Furthermore, evidence suggests that melanin in the fungal cell wall may contribute to enhancing host plant resilience. Additionally, melanin in DSEs has been demonstrated to increase the mechanical strength of the cell wall, protect DSE hyphae under drought conditions, and safeguard organelles to ensure that photosynthesis occurs (Zuo et al., 2022).

In response to drought-induced osmotic stress, osmotic adjustment represents a crucial mechanism through which plants regulate water potential, thereby enabling them to adapt to drought stress (Hanif et al., 2021). Pro, SS and SP are highly sensitive to alterations in soil water levels and are capable of reducing the cellular osmotic potential when plants are subjected to drought stress. The accumulation of these compounds is frequently employed as a marker of drought resistance in plants (Liu F. et al., 2023). The results of this study indicate that the contents of Pro and SS increased rapidly in plants subjected to drought stress. This suggests that *C. dactylon* can increase the contents of Pro and SS through its own metabolic processes, thereby enabling plant cells to retain water and preventing the normal development of plants from being affected by dehydration. Notably, the change in SP content observed in this study differed from the findings of Wang et al. (2019) who reported that the SP content increased with increasing drought intensity. In this study, there was a significant reduction in the SP content under severe drought stress. This discrepancy may be attributed to the loss of proteins due to severe damage to the cell membrane under severe drought stress, with some of the proteins being diverted for cell membrane repair (Liu H. et al., 2022). The contents of Pro, SS, and SP in *C. dactylon* were significantly increase following DSE inoculation in this study. Notably, the increase in the plant SP content caused by DSE inoculation was more pronounced under severe drought stress. These findings indicate that DSE inoculation can facilitate the accumulation of osmoregulatory substances in plants and reduce the osmotic potential of host cells, thereby mitigating the dehydration loss caused by drought stress and further promoting the response of plant defense mechanisms against drought stress.

The cell membrane is a crucial interface between plant cells and organelles and the surrounding environment. It plays a pivotal role in maintaining cellular stability and regulating material exchange, energy flow and information transfer (Zhang H. et al., 2022). The tolerance of plants to stress is closely related to the stability of the biofilm structure and its function (He et al., 2020). Drought stress frequently results in the extravasation of organic molecules and electrolytes within cells, which in turn exacerbates membrane lipid peroxidation and ultimately leads to destruction of the structure and integrity of plant cell membranes (Wang et al., 2023). MDA which is produced because of cellular membrane lipid peroxidation, provides a measure of the extent of damage to plant cells caused by adversity (Batool et al., 2020). The results indicated that MDA content increased in direct proportion to the severity of drought stress, reinforcing the hypothesis that severe drought stress leads to significant damage to cell membranes. Conversely, the MDA content of *C. dactylon* inoculated with DSE under drought conditions exhibited a notable decline compared to that of *C. dactylon* in the CK. These findings suggest that treatment with DSE may mitigate the accumulation of MDA in *C. dactylon* and reduce the formation of reactive oxyradicals, thereby limiting the extent of cell membrane lipid peroxidation and mitigating the adverse effects of drought stress on *C. dactylon.*

When plants experience drought stress, elevated levels of reactive oxygen species (ROS) are typically produced; these ROS contribute to the breakdown of lipids, proteins, and nucleic acids within plant cells. Furthermore, ROS function as pivotal signaling molecules throughout the cell death pathway (Liu N. et al., 2022). The antioxidant system is crucial for plants and microorganisms to cope with oxidative damage (Li X. et al., 2022). A substantial accumulation of ROS activates antioxidant enzymes in plants, with various enzymes acting in concert to safeguard plants from stress (Akhtar et al., 2022). Furthermore, some scholars have demonstrated that endophytes can increase antioxidant enzyme activities in plants by influencing gene expression during stress and increasing the capacity of plants to withstand adverse stress (Zhang Y. et al., 2022). The findings of the present study indicate that the antioxidant enzyme activities in plants first increased, followed by a subsequent decrease in response to an increase in the degree of drought stress. SOD is considered a crucial enzyme in the scavenging of reactive oxyradicals. Specifically, plant SOD activity was elevated in response to the DSE treatments, potentially due to the promotion of its synthesis by DSEs. This facilitates the conversion of the more toxic O_2_^·−^ to the less toxic H_2_O_2_. The observed increase in SOD activity may, therefore, positively influence the conversion of ROS to H_2_O_2_ in plants (Singh et al., 2020). The inoculation of DSEs under drought stress increased the activities of POD and CAT in the plants. This may be attributed to the fact that H_2_O_2_ produced by the disproportionation of ROS by SOD requires further conversion into non-toxic H_2_O and O_2_ by the synergistic action of the more active POD and CAT (Song et al., 2024). These findings suggest that the excess ROS produced by the plants under drought stress were effectively scavenged, alleviating the drought-induced membrane lipid peroxidation of *C. dactylon* cells. Therefore, treatment with DSEs helped the plants to increase their antioxidant enzyme activities *in vivo* and maintain them at high levels, effectively scavenging the excess ROS produced by drought stress in the plant and ensuring the stability and integrity of the cell membrane.

According to the results of principal component analysis and correlation analysis, the photosynthesis, osmoregulation and antioxidant enzymes of plants play significant roles in resistance to adverse stress. When *C. dactylon* is subjected to drought stress, increased photosynthesis positively promotes the accumulation of *C. dactylon* biomass. Moreover, drought stress activated the antioxidant mechanism in *C. dactylon* and further increased the activity of antioxidant enzymes after inoculation with DSEs, which helped eliminate the excessive amounts of ROS produced due to drought stress. In addition, the accumulation of osmotic adjustment substances such as Pro, SS and SP helps maintain the imbalance of osmotic pressure inside and outside the cells caused by drought and reduces lipid peroxidation of cell membranes. The coordinated work of various systems in plants helps *C. dactylon* resist drought stress and maintain its normal growth.

## 5 Conclusion

The objective of this study was to evaluate the impacts of three distinct DSEs on the growth of *C. dactylon* in vegetation concrete subjected to drought stress. Despite the inhibitory effects of drought stress on the normal physiological mechanisms of *C. dactylon*, which resulted in a reduction in both plant height and dry weight, *C. dactylon* exhibited robust drought tolerance when inoculated with DSE. Compared with those of the CK, the Pn, Gs and Tr of *C. dactylon* increased by 33.85%, 46.17% and 33.64%, respectively, following inoculation with DSE. Compared with the uninoculated plants, *C. dactylon* plants in the treatments with DSE strains had a greater accumulation of osmoregulatory substances and higher antioxidant enzyme activities. Consequently, DSE has the capacity to mitigate the adverse effects of drought on *C. dactylon* in vegetation concrete to a certain extent and has the potential for further development in the application of ecological restoration technology for vegetation concrete slopes under adverse conditions.

## Data Availability

The raw data supporting the conclusions of this article will be made available by the authors, without undue reservation. Requests to access these datasets should be directed to ML, limingyi@ctgu.edu.cn.
